# Beyond Borders and Genders: Unveiling Cultural Influences on Badminton Motivation Among Older Adult Players in Taiwan and the United States

**DOI:** 10.3390/sports12110313

**Published:** 2024-11-20

**Authors:** Wei-Chieh Liao, Yun-Dih Chia-Smith, David Cabello-Manrique, Chia-Min Wang, Li-An Liao

**Affiliations:** 1 Department of Ball Sports, University of Taipei, Taipei 11153, Taiwan; king.liao@yahoo.com.tw; 2 School of Education, Loyola University Maryland, Baltimore, MD 21210, USA; ychiasmith@loyola.edu; 3 Department of Physical Education and Sports, University of Granada, 18011 Granada, Spain; dcabello@ugr.es; 4 Office of Physical Education, Soochow University, Taipei 111002, Taiwan; wcm917001@hotmail.com

**Keywords:** successful aging, older in sports, culture/gender comparison, health promotion, badminton

## Abstract

This study explores and compares the motivations behind badminton participation among older adult players from Taiwan and the United States (U.S.), with a specific focus on cultural and gender differences. A total of 139 participants aged 60 and above took part in the study, with 55 from the United States (24 females, 31 males) and 84 from Taiwan (39 females, 45 males). Utilizing the Exercise Motivations Inventory-2 (EMI-2), this research examines differences in motivations across cultural and gender lines, identifying key factors such as health-related motivations, personal achievement, social interactions, and competition. The results reveal that Taiwanese players are more motivated by health benefits and stress reduction, while U.S. players emphasize personal achievement and recognition. Additionally, gender-specific motivations emerged, with female players from both countries placing greater importance on social interactions compared to their male counterparts. These findings underscore the need for culturally and gender-sensitive approaches to promote sports participation among older adults in diverse settings.

## 1. Introduction

The global population is getting older, posing challenges and opportunities for health and individual well-being. As people live longer, staying active and leading a fulfilling life becomes crucial for improving the quality of life among the older. Being physically active is key to maintaining health and well-being in adults, helping to reduce the risks linked to chronic diseases and cognitive decline [1,2,3,4]. Sport fosters physical health and contributes to mental wellness through its social aspects and the cognitive demands of game strategy [5,6,7]. Among various sports, badminton stands out as particularly suitable for older adults due to its low-impact, non-contact nature, which minimizes injury risk. Its adaptability to various skill levels makes it accessible, while its social and strategic elements enhance mental and social well-being, making it an excellent choice for seniors [8].

Understanding what motivates older adults to participate in sports like badminton is crucial for developing effective health promotion programs. Motivation in older sports participation is influenced by various factors, including the pursuit of health benefits, social interactions, and personal enjoyment [9]. According to Self-Determination Theory (SDT), these motivations are often fulfilled when the activities meet intrinsic psychological needs related to autonomy, competence, and relatedness [10].

This study focuses on Taiwan and the United States (U.S.), chosen for their contrasting cultural values: Taiwan’s collectivist orientation encourages communal values and social recognition in sports, while the U.S.’s individualistic culture promotes personal achievement and self-improvement [11,12]. Studying these two countries provides a valuable perspective on how different cultural frameworks influence sports motivation, especially among older adults. Such a cross-cultural comparison will allow us to develop targeted approaches that align with the specific cultural values and needs of different communities [13].

Gender differences for individuals participating in sports are influenced by a mix of psychological, social, and cultural aspects. Previous study showed that men are often driven by competition and personal achievement, while women tend to prioritize the connections and stress relief that sports offer [14]. Men’s lifelong involvement in sports typically leads them to seek competition and physical improvement as they age [7,15]. On the other hand, older women may see sports as a way to build relationships and find support, especially if societal norms limit their participation earlier in life [16,17,18]. Additionally, compared to men, women’s motivation can be affected by self-confidence and their perceived athletic abilities [19,20].

While this study includes gender-based motivations, the primary focus is on cultural differences in sports motivation between Taiwan and the U.S. This approach aligns with the study’s objective to emphasize the influence of cultural context, with gender differences considered to provide additional insights into creating inclusive sports programs that resonate with diverse populations [21].

The purpose of this study is to explore and compare the motivations of older adults badminton players in Taiwan and the United States. It targeted particular focus on gender and cultural differences in their participation in badminton. Examining what drives senior athletes to participate in badminton, the research investigates the distinct and shared motivational factor across these two cultural groups. Understanding the differences and similarities will provide insights into how cultural context and gender roles influence sports participation among the older. This knowledge helps design the targeted programs and policies promoting physical activity and well-being among older adults in different cultural settings.

## 2. Materials and Methods

### 2.1. Participants

A total of 139 older adult badminton players, recruited from various badminton clubs in the U.S. and Taiwan, voluntarily participated in this study.

To ensure the adequacy of the sample size, a power analysis was conducted using G*Power software 3.1.9.7 (Heinrich-Heine-Universität Düsseldorf, Düsseldorf, Germany). With an anticipated medium effect size (f = 0.25), an alpha level of 0.05, and a desired power of 0.80, the analysis indicated that a minimum of 128 participants would be required. Our sample of 139 participants meets and exceeds this requirement, providing confidence in the validity and robustness of our findings.

Participants were recruited from community and recreational badminton clubs in both Taiwan and the U.S. This method was chosen to capture a representative sample of older recreational badminton players who actively engage in the sport. Club coordinators facilitated the recruitment process, inviting members who met the age criteria and engaged in regular play. This approach helps ensure that our sample reflects the broader population of older adults badminton players in each region.

Inclusion criteria required participants to be at least 60 years old, actively engaged in recreational badminton, and willing to participate voluntarily. The recruitment process emphasized voluntary self-selection, with club coordinators providing general health guidance, ensuring that participants were capable of safe participation in sports activities.

U.S. Sample: Fifty-five recreational badminton players, aged between 60 and 74 years (M = 64.98, SD = 4.51), participated. The sample comprised 24 females (M = 64.88, SD = 4.94) and 31 males (M = 65.06, SD = 4.24). All participants were non-smokers. Employment status revealed that 5.45% were part-time, 50.91% were full-time, and 43.64% were retired (part-time: 3, full-time: 28, retired: 24). A majority (98.19%) identified as right-handed, with only one left-handed participant. Marital status was diverse: 7.27% were single, 1.82% were widowed, and 90.91% were married (singles: 4, widowed: 1, and married: 50). Living arrangements showed that three participants lived independently, while the remainder resided with family. Experience levels varied, with years of play ranging from 12 to 55 years (M = 31.03, SD = 11.07). Participants began engaging in recreational badminton at an average age of 33.96 years (SD = 11.24), with an average Body Mass Index (BMI) of 22.39 (SD = 2.63).

Taiwanese Sample: This group consisted of 84 individuals aged 60 to 72 years (M = 67.02, SD = 4.69), including 39 females (M = 65.08, SD = 5.29) and 45 males (M = 68.71, SD = 3.32). Among them, two reported being smokers. Employment status indicated that 9.52% were part-time, 15.48% were full-time, and 75% were retired (part-time: 8, full-time: 13, retired: 63). The majority 99.37% were right-handed, with only one left-handed participant. In terms of marital status, 1.19% were single, 1.19% were widowed, 92% were married, and 4.76% were N/R (single: 1, widowed: 1, married: 78, and no responses: 4). Their playing experience ranged from 4 to 45 years (M = 23.43, SD = 10.19), starting at an average age of 43.96 years (SD = 9.5). The average BMI was 24.39 (SD = 2.63).

Body Mass Index (BMI) was calculated for all participants using the standard formula BMI = Weight (kg)/[Height (m)]^2^, providing a consistent measure of physical health across both groups. Demographic characteristics, including age, height, weight, employment status, and years of playing experience, are summarized in Table 1 to ensure clarity and allow for comparisons between the U.S. and Taiwanese samples.

### 2.2. Study Design

#### 2.2.1. Instruments

The study employed two main instruments:

- Demographic Questionnaire: Participants provided demographic details, including age, height, weight, and badminton-related information such as frequency and duration of play per week, and years of experience.
- Exercise Motivations Inventory-2 (EMI-2): This inventory assessed the motivation behind participating in badminton, based on Deci and Ryan’s self-determination theory. The EMI-2 demonstrates acceptable to high reliability, with Cronbach’s alpha values ranging from 0.71 to 0.91, supporting its use across diverse populations [22]. The EMI-2 consists of 51 items covering seven main subscales: stress management, revitalization, enjoyment, challenge, social recognition, affiliation, competition, and four additional subscales focused on health and appearance motivations. Responses were recorded on a six-point Likert scale (1 indicated ‘not at all true for me’ and 6 indicated ‘very true for me’). To ensure accuracy in cross-cultural contexts, the EMI-2 was translated into Chinese through a “forward-backward” translation process, involving bilingual experts and a pilot test with Taiwanese college students fluent in English.

#### 2.2.2. Procedure

Questionnaire Administration: The questionnaires were administered in a controlled setting at each badminton club, allowing participants to respond in a familiar environment. A trained research assistant was available to answer questions and ensure the clarity of instructions, maintaining consistency in data collection across both countries. The average completion time for the questionnaires was approximately 20 min.

#### 2.2.3. Data Analysis

Software and Statistical Methods: Data analysis was conducted using IBM SPSS Statistics for Windows, Version 29.0.2.0 Armonk, NY, USA: IBM Corp. Descriptive statistics, including means, standard deviations, and percentages, were calculated to summarize demographic and motivational data. Independent t-tests and ANOVA were employed to examine differences in motivational factors across cultural and gender groups, with a significance level set at 0.05. Effect sizes were calculated to provide insights into the practical significance of findings.

## 3. Results

### 3.1. Playing Experience

The study revealed significant differences in playing experience between U.S. and Taiwanese badminton players. American players reported an average of approximately 31.03 years of experience in playing badminton, markedly higher than the average of around 23 years for Taiwanese players, with the difference being statistically significant (*p* < 0.05). Furthermore, U.S. participants generally began playing badminton at a younger age, with an average start age of 33.96 years, in contrast to 43.96 years for Taiwanese participants, indicating a significant difference (*p* < 0.001). This disparity in playing experience suggests that U.S. players may have a cultural predisposition towards early sports involvement, potentially due to greater emphasis on individual sports activities from a young age, aligning with the individualistic values prevalent in the U.S.

### 3.2. Motivation for Participation

#### 3.2.1. Between Taiwanese and Americans

Both U.S. and Taiwanese players ranked enjoyment, affiliation, positive health, nimbleness, and revitalization as their top five motivations for participating in badminton, albeit in slightly different orders. Taiwanese players exhibited higher motivation across all subscales, with significant differences observed in nine out of 14 subscales, including stress reduction, interest in challenge and competition, social recognition, health-related motivations, physical appearance enhancement, weight management, and strength endurance improvement.

Cultural Differences in Motivation: The higher motivation among Taiwanese players for stress reduction (F = 28.237, *p* < 0.001), challenge (F = 14.195, *p* < 0.001), and competition (F = 11.77, *p* < 0.01) reflects the collectivist nature of Taiwanese society, where sports are often valued as tools for social harmony, stress management, and structured competition. In contrast, U.S. players, guided by individualistic cultural values, showed a stronger focus on personal achievement and skill comparison, indicating a drive for individual recognition over communal approval (Table 2).

#### 3.2.2. Between Male and Female Players

The investigation into gender differences revealed that both female and male players identified affiliation, positive health, enjoyment, revitalization, and nimbleness as their primary motivations, though in varying orders. Females displayed stronger overall motivation, in particular in stress management, goal setting, skill enhancement, and social interactions. The stronger emphasis on social factors, such as “spending time with friends” and “enjoying social aspects”, was more pronounced in female participants, reflecting gender norms in both societies that align women’s sports involvement with socializing and emotional support (Table 3).

### 3.3. Intersection of Sex and Region on Motivation

**Figure 1:**
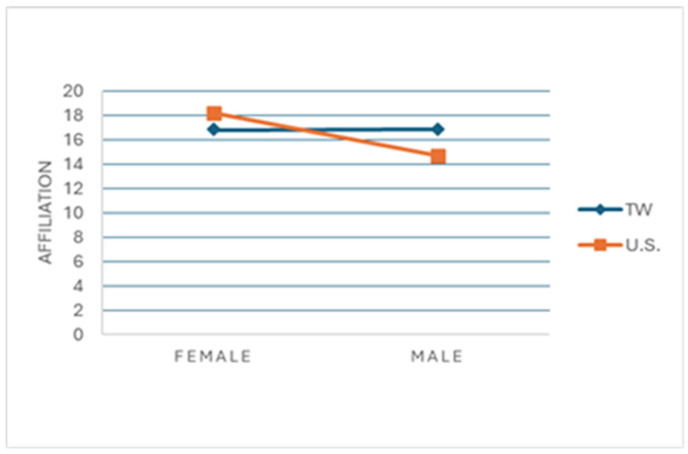
Interaction between sex and region on motivation for social participation among badminton players.

The analysis further identified a significant effect of sex on motivation, particularly in the domain of ‘Affiliation’, where females showed greater motivation for social participation (F = 7.45, *p* < 0.01). This interaction between sex and region (F = 7.86, *p* < 0.01) (Figure 1) revealed that U.S. women prioritize social interaction more than Taiwanese women, likely due to cultural differences: the individualistic culture in the U.S. may encourage communal sports as a counterpoint to societal norms, whereas Taiwanese culture, with its collectivist values, provides social networks beyond sports, resulting in a less pronounced need for social affiliation through sports.

## 4. Discussion

This comprehensive study explores the complex motivational factors that drive older adult badminton players participation. It highlights the significant impacts of cultural contexts, gender differences, and individual experiences. These factors play crucial roles in shaping their engagement with the sport. Significant disparities in playing experiences between American and Taiwanese players have emerged. Americans typically engage with badminton over extended periods (averaging 31.03 years), suggesting a cultural norm involving early sports initiation and sustained engagement, potentially supported by family and organized youth sports [1,9]. Taiwanese players, conversely, begin at later stages, possibly due to different cultural attitudes toward sports participation.

### 4.1. Sociocultural Influences on Sports Motivation

American and Taiwanese players share core motivations for playing badminton, such as enjoyment and health, but the emphasis placed on each varies, reflecting distinct cultural values. Taiwanese participants, consistent with a collectivist cultural orientation, exhibited higher motivation for stress management, competition, and social recognition compared to their U.S. counterparts [11,12]. This aligns with other research findings in sports like Tai Chi and community fitness, where collectivist societies often emphasize health benefits and social engagement as key motivators [23]. In Taiwan, social networks and the healthcare system significantly shape these motivations, with family expectations and medical advice playing crucial roles in encouraging sports participation [23].

On the other hand, U.S. players, influenced by individualistic cultural values, were more motivated by personal achievement and self-improvement, trends seen in Western sports such as running and swimming [24]. This reflects the U.S. cultural emphasis on individualism, where personal achievement often takes precedence over social recognition [1]. For American players, badminton is more commonly viewed as a recreational or social activity rather than a primary fitness method, which illustrates distinct attitudes toward physical appearance and body image across cultures. Taiwanese players, however, place greater importance on health-related areas, such as stress reduction and physical well-being, reflecting a broader cultural valuation of communal health [10,14]. These differences highlight broader societal influences on how sports are valued, suggesting that motivational patterns are deeply embedded in cultural values, reinforcing the need for culturally sensitive program designs.

Given these cultural distinctions, developing badminton programs that align with each culture’s unique motivations is crucial. In Taiwan, where health-related motivations are prominent, programs could highlight badminton’s health benefits and integrate them into broader community wellness initiatives [16]. In contrast, U.S. programs may be more effective if they focus on personal achievement and offer competitive opportunities, resonating with American values [20]. This culturally sensitive approach, informed by the unique motivational drivers of each group, can enhance player engagement and promote sports participation among older adults across diverse cultural backgrounds.

### 4.2. Gender-Specific Motivational Differences

Gender-based differences in motivation are evident among badminton players, with female players showing stronger overall motivation, particularly for stress management and social interactions. This suggests that women place significant value on the communal and therapeutic aspects of sports, aligning with previous findings on gendered motivations in physical activity [23]. Programs aiming to increase badminton participation should consider these gender-specific motivations, potentially offering more social and stress-relief components to appeal to female participants [1,14]. For instance, health-related motivations, such as positive health and physical appearance, were more prominent among female players, indicating that health promotion programs could be particularly appealing to older women. However, it is essential to ensure these programs promote a healthy body image without overemphasizing appearance [16,20].

For male participants, who are often driven by competition and skill development, programs emphasizing these elements may be more effective, supporting a gender-sensitive approach to sport promotion. While female players tend to value stress management, men might not perceive the same mental health benefits from badminton, potentially due to cultural norms that discourage men from using sports as a stress-management tool. Addressing these barriers could make sports programs more effective for male participants, as competition and skill enhancement may resonate more effectively with their motivations.

The importance of social factors also varies, with social interactions being particularly significant for women, which challenges the assumption that men are generally more socially driven in sports. This finding suggests the need for creating social environments in sports programs that cater to women’s preferences, enhancing the communal aspects that they find motivating. Recognizing and addressing these gender-specific motivations can lead to more inclusive and effective sports programs, ultimately promoting better health and well-being among older adults.

### 4.3. The Dual Impact of Gender and Culture on Motivation

The interaction between gender and cultural background adds another layer of complexity to motivational factors. Affiliation motivation, for example, was particularly strong among U.S. women who may find communal sports appealing in contrast to the individualistic norms prevalent in the U.S. In Taiwan, the collectivist culture may already provide social networks that fulfill these needs outside of sports, resulting in a less pronounced gender difference in sports motivations. These insights reveal how gender and cultural contexts collectively shape motivation, underscoring the need for tailored sports programs that resonate with specific cultural and gender dynamics.

### 4.4. Comprehensive Nature of the Study

This study can be considered comprehensive due to its depth of analysis and comparative approach across two distinct cultural contexts and both genders. By examining multiple motivational factors among players in Taiwan and the U.S., this research provides a holistic understanding of sports motivation encompassing cultural and gender-based variations. Utilizing the Exercise Motivations Inventory-2 (EMI-2) allowed an in-depth exploration of 14 motivational subscales, including health, social, and personal achievement dimensions. This level of detail, rarely seen in similar studies, contributes valuable insights into older adult sports participation motivations, making a significant impact on both cultural and gender perspectives.

### 4.5. Implications for Intervention Strategies

These findings are invaluable for designing cultural and gender-sensitive interventions that promote sustained engagement in badminton among older adults. In Taiwan, where health motivations prevail, programs should collaborate with healthcare entities to emphasize badminton’s health benefits and integrate it into broader community wellness initiatives. In the U.S., interventions focusing on personal achievement and offering competitive opportunities may better align with individualistic cultural values. For women, organizing women-only sessions or combining play with social events could provide an inviting atmosphere that caters to their social motivations, effectively promoting active aging through badminton.

### 4.6. Limitations and Future Directions

Although our sample size met the statistical power requirements, limitations related to representativeness should be acknowledged. The sample’s majority from Taiwan may limit the findings’ generalizability, especially regarding broader older adult populations in each country. Additionally, the age range (M = 64.98, SD = 4.51) does not capture the entire older adult demographic, potentially limiting applicability to older subgroups. Future research should aim for larger and more diverse samples to enhance generalizability. Future research could include additional cultural contexts to enhance the generalizability of findings and provide a more comprehensive understanding of how diverse cultural backgrounds impact sports motivation among older adults. Moreover, reliance on self-reported measures introduces potential cultural response biases. Longitudinal and qualitative studies could provide deeper insights into how cultural influences, gender differences, and individual experiences affect motivations over time.

## 5. Conclusions

This research highlights the importance of cultural contexts and gender differences in shaping motivation for badminton participation among older adults, specifically within the Taiwanese and U.S. populations. These findings call for culturally sensitive and gender-inclusive strategies to promote active and healthy lifestyles in a way that aligns with the unique motivational drivers identified in each group. While badminton’s appeal is broad and age-friendly, motivational differences across cultures and genders emphasize the need for targeted strategies that directly address cultural values and gender-specific factors relevant to motivation in sports. As badminton’s popularity continues to grow and societies age, these insights will be crucial for developing inclusive, effective programs that support sustained participation and well-being in diverse populations.

## Figures and Tables

**Table 1:** Demographic and recreational characteristics of older U.S. and Taiwanese adult badminton players.

	U.S. Sample (*n* = 55; 31 Males, 24 Females)			Taiwanese Sample (*n* = 84; 45 Males, 39 Females)		
	Mean	SD		Mean	SD	
Age, overall (years)	64.98	4.51		67.02	4.69	
Age, male (years)	65.06	4.24		68.71	3.32	
Age, female (years)	64.88	4.94		65.08	5.29	
Playing Experience (years)	31.01	11.07		23.43	10.19	
Starting Age for Badminton	33.96	11.24		43.96	9.5	
Body Mass Index (BMI)	24.39	2.63		24.11	2.11	
Height (cm)	165.9	10.10		165.01	11.72	
Weight (kg)	66.96	12.19		65.63	10.68	
Smoking Status			All non-smokers			2 smokers
Employment Status			5.45% part-time, 50.91% full-time, 43.64% retired			9.52% part-time, 15.48% full-time, 75% retired
Handedness			98.19% right-handed 1 left-handed			99.37%, right-handed 1 left-handed
Marital Status			7.27% single, 1.82% widowed, 90.91% married			1.19% single, 1.19% widowed, 92% married, 4.76% N/R

**Table 2:** Motivation scale comparisons.

Scales	U.S. (*n* = 55)	Taiwan (*n* = 84)	F	Sig.
	Mean (SD)	Mean (SD)		
Total score	154.36 (5.38)	188.09 (4.32)	23.859 **	0.000
Revitalization	11.26 (0.334)	12.27 (0.269)	5.572 *	0.020
Stress Management *	10.63 (0.621)	14.87 (0.449)	28.237 **	0.000
Enjoyment	16.59 (0.410)	16.34 (0.330)	0.237	0.627
Challenge *	11.37 (0.667)	14.60 (0.536)	14.195 **	0.000
Social Recognition *	9.5 (0.658)	11.85 (0.529)	7.693 **	0.006
Affiliation	16.44 (0.487)	16.84 (0.392)	0.421	0.517
Competition *	10.79 (0.675)	13.76 (0.543)	11.777 **	0.001
Health Pressures *	4.69 (0.474)	8.12 (0.381)	31.840 **	0.000
Ill-health Avoidance *	9.37 (0.459)	11.40 (0.369)	11.944 **	0.001
Positive Health	11.94 (0.387)	12.92 (0.311)	3.949	0.049
Weight Management *	8.16 (0.691)	13.30 (0.556)	35.512 **	0.000
Appearance *	8.92 (0.676)	13.72 (0.544)	30.649 **	0.000
Strength and Endurance *	13.40 (0.606)	15.53 (4.88)	7.488 **	0.007
Nimbleness	11.50 (0.337)	12.49 (0.304)	4.211 **	0.042

* = Significant difference at *p* < 0.05 ** = Significant difference at *p* < 0.01 F = F-statistic from ANOVA test.

**Table 3:** Mean and standard deviation comparison on motivation subscale by sex.

Scales	Female (*n* = 63)	Male (*n* = 76)	F	Sig.
	Mean (SD)	Mean (SD)		
Total score *	180.73 (5.13)	161.73 (4.61)	7.57 **	0.007
Revitalization	12.09 (0.319)	11.43 (0.287)	2.30	0.131
Stress Management *	13.91 (0.592)	11.59 (0.533)	8.45 **	0.004
Enjoyment	16.93 (0.392)	15.99 (0.352)	3.15	0.078
Challenge *	14.16 (0.636)	11.81 (0.572)	7.58 **	0.007
Social Recognition	11.39 (0.628)	9.96 (0.565)	2.87	0.092
Affiliation *	17.49 (0.464)	15.79 (0.418)	7.45 **	0.007
Competition	12.93 (0.644)	11.61 (0.580)	2.33	0.129
Health Pressures	7.09 (0.452)	5.73 (0.407)	5.03 **	0.027
Ill-health Avoidance	10.47 (0.438)	10.35 (0.394)	0.078	0.780
Positive Health	13.01 (0.369)	11.84 (0.332)	5.60 **	0.019
Weight Management	11.43 (0.660)	10.03 (0.593)	2.53	0.114
Appearance	12.33 (0.645)	10.31 (0.580)	5.42 **	0.022
Strength and Endurance	14.94 (0.578)	13.99 (0.520)	1.49	0.224
Nimbleness	12.46 (0.360)	11.54 (0.324)	3.59	0.060

* = Significant difference at *p* < 0.05 ** = Significant difference at *p* < 0.01 F = F-statistic from ANOVA test.

## Data Availability

The data that support the findings of this study are available from the corresponding author upon reasonable request.
